# Cellular and molecular mechanisms of immunomodulation in the brain through environmental enrichment

**DOI:** 10.3389/fncel.2014.00097

**Published:** 2014-04-03

**Authors:** Gaurav Singhal, Emily J. Jaehne, Frances Corrigan, Bernhard T. Baune

**Affiliations:** ^1^Psychiatric Neuroscience Lab, Discipline of Psychiatry, School of Medicine, University of AdelaideAdelaide, SA, Australia; ^2^Discipline of Anatomy and Physiology, School of Medical Sciences, University of AdelaideAdelaide, SA, Australia

**Keywords:** environmental enrichment, immune, cytokines, glial cells, T cells, neurobiology, cognition, behavior

## Abstract

Recent studies on environmental enrichment (EE) have shown cytokines, cellular immune components [e.g., T lymphocytes, natural killer (NK) cells], and glial cells in causal relationship to EE in bringing out changes to neurobiology and behavior. The purpose of this review is to evaluate these neuroimmune mechanisms associated with neurobiological and behavioral changes in response to different EE methods. We systematically reviewed common research databases. After applying all inclusion and exclusion criteria, 328 articles remained for this review. Physical exercise (PE), a form of EE, elicits anti-inflammatory and neuromodulatory effects through interaction with several immune pathways including interleukin (IL)-6 secretion from muscle fibers, reduced expression of Toll-like receptors on monocytes and macrophages, reduced secretion of adipokines, modulation of hippocampal T cells, priming of microglia, and upregulation of mitogen-activated protein kinase phosphatase-1 in central nervous system. In contrast, immunomodulatory roles of other enrichment methods are not studied extensively. Nonetheless, studies showing reduction in the expression of IL-1β and tumor necrosis factor-α in response to enrichment with novel objects and accessories suggest anti-inflammatory effects of novel environment. Likewise, social enrichment, though considered a necessity for healthy behavior, results in immunosuppression in socially defeated animals. This has been attributed to reduction in T lymphocytes, NK cells and IL-10 in subordinate animals. EE through sensory stimuli has been investigated to a lesser extent and the effect on immune factors has not been evaluated yet. Discovery of this multidimensional relationship between immune system, brain functioning, and EE has paved a way toward formulating environ-immuno therapies for treating psychiatric illnesses with minimal use of pharmacotherapy. While the immunomodulatory role of PE has been evaluated extensively, more research is required to investigate neuroimmune changes associated with other enrichment methods.

## INTRODUCTION

Cognitive deficit, memory loss, and behavioral impairment underpin most psychiatric disorders. Several etiologies such as age, gender, and race (Piccinelli and Wilkinson, 2000; Gottlieb et al., 2004; Hedden and Gabrieli, 2004), stress (Lupien et al., 2009), socioeconomic status (Gilman et al., 2002; Lorant et al., 2003), metabolic disorders (Simon et al., 2006; Rinaldi et al., 2014), gene–environment interactions (Caspi and Moffitt, 2006), and neuroinflammation (Campbell, 2004; Ownby, 2010; Tansey and Goldberg, 2010) have been implicated for the impairment of brain function. Contrary to this, environmental enrichment (EE), a concept of “modifying the environment of captive animals to enhance their physical and psychological well-being by providing stimuli meeting their species-specific need” (Baumans, 2005), has been shown to slow down neuronal aging (Gould et al., 2000; Kempermann et al., 2002) and improve cognition, memory, behavior, and motor coordination in pre-clinical models of dementia, depression, Alzheimer’s disease (AD), Parkinson’s disease (PD), and Huntington’s disease (Faherty et al., 2005; Jankowsky et al., 2005; Hannan, 2014).

Although most EE paradigms used rodents for research, other animals like rabbits (Hansen and Berthelsen, 2000), pigs (van de Weerd and Day, 2009), fish (Batzina et al., 2014), and primates (e.g., marmosets; Kozorovitskiy et al., 2005) have also been used in EE studies. Researchers have used several methods of EE for rodents in cages, either alone or in combination. Physical exercise (PE), social housing, and enrichment with novel objects and accessories are the most commonly used methods. On the other hand, the sensory method of enrichment, where a sensory stimulus such as visual, auditory, and olfactory stimuli is given to stimulate brain functions, has been used to a lesser extent.

Equivalent treatments of EE in rodents can be seen in human literature. Although not operationally similar, these treatments promote mental stimulation and can provide enrichment to the standard human environment, similar to EE in rodent studies. These could include aerobic exercise (Colcombe et al., 2006; Hillman et al., 2008), an active and socially integrated lifestyle (Fratiglioni et al., 2004), cognitive training with brain storming exercises (Willis et al., 2006; Miller et al., 2013), learning of complex tasks (e.g., learning to juggle balls; Draganski et al., 2004; Boyke et al., 2008), extensive learning during examinations (Draganski et al., 2006), food supplementation (Fuglestad et al., 2008), and sensory enhancement (e.g., listening to favorite music; Särkämö et al., 2008; Koelsch, 2010). Like EE in rodents, these treatments have shown similar effects in humans, improving cognition and memory, which means there may be similar mechanisms of action of both on the central nervous system (CNS).

Recent studies have shown that EE is able to affect cytokines, various immune components and glial cells suggesting this may be a potential mechanism of action for how it may modulate brain function. The discovery of the modulation of neuroimmune mechanisms by EE has provided a potential mechanism of action of this intervention. Circulating immune cells and proteins (e.g., T cells and cytokines) maintain the brain homeostasis (Ron-Harel et al., 2011) forming a bi-directional neuroimmune pathway which can affect behavior, mood, and cognition (Maier and Watkins, 1998). It has been suggested that there are modifications in several immune markers, for example, cytokines (Pedersen and Hoffman-Goetz, 2000), chemokines (Trøseid et al., 2004), T cells (Marashi et al., 2003, 2004), natural killer (NK) cells (Benaroya-Milshtein et al., 2004), Toll-like receptors (TLRs; Gleeson et al., 2006), C-reactive protein (CRP; Koletzko, 2003), and glial cells (Ehninger and Kempermann, 2003; Williamson et al., 2012) alongside neurobiological and behavioral alterations in rodents treated with different enrichment techniques.

Regardless of this significant relationship, the immunomodulatory role of EE has received less attention than its neurobiological and behavioral effects. While independent rodent studies predominantly put forward a role of PE in inducing changes to neuroimmune markers such as cytokines [e.g., tumor necrosis factor-α (TNF-α), interleukin (IL)-1β, IL-6], T lymphocytes, NK cells, glial cells (e.g., microglia), CRP, and the complement system, less information is available about the immunomodulatory role of other enrichment methods, like social housing and enrichment with novel objects and accessories in rodents. In this review, we aim to fill this gap in the existing literature by conducting a critical analysis of randomized controlled trials (RCTs) on rodents and analogous human studies, exploring the neuroimmune modulatory effects of EE and how this may improve cognition and memory. A particular emphasis is placed on how neuroimmune mechanisms that modulate brain function differ in response to various EE methods in this review.

## MATERIALS AND METHODS

### PRISMA CRITERIA

We followed the guidelines prescribed by PRISMA (preferred reporting items for systematic reviews and meta-analyses; Liberati et al., 2009; Moher et al., 2009) while constructing this review. The checklist items from PRISMA as relevant to this review, for example, those related to search and writing approaches, were included and the items not relevant, for example, those related to meta-analyses, were excluded.

### SEARCH AND SELECTION PROCESS

**FIGURE 1:**
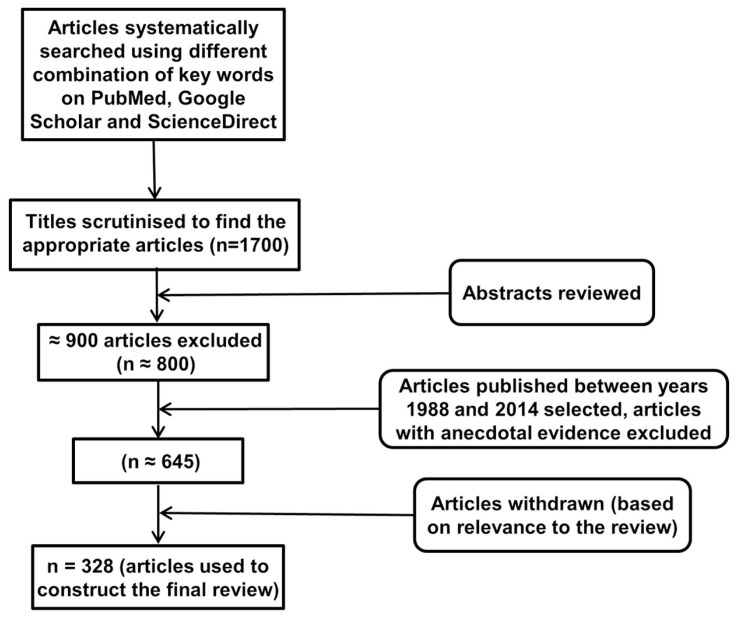
**Study inclusion flowchart.** It depicts the methodology for search and collection of relevant articles for this review, following PRISMA guidelines (Liberati et al., 2009; Moher et al., 2009).

An electronic database search of PubMed, Google Scholar, and ScienceDirect with the following key terms in various permutations was performed: environmental enrichment, immune, neuroimmune, cytokine, glial cells, T cells, B cells, immunoglobulins, NK cells, Toll-like receptor, C-reactive protein, complement system, neuroplasticity, neuropathology, inflammation, neuroinflammation, cognition, cognitive stimulation, cognitive remediation, cognitive training, cognitive rehabilitation, memory, behavior, physical exercise, novelty, rodents, social enrichment, social interaction, social factors, cage enrichment, nesting, nutrition, sensory enrichment, visual cortex, auditory cortex, olfactory bulb, music, and motor activity. At each stage of the search, titles and abstracts were scrutinized and the most appropriate organized into separate folders using End Note X6.0.1 software. In addition, articles relevant to our discussion were retrieved from the reference list of other online articles on each subtopic. This in total yielded approximately 1700 papers. After placing all inclusion and exclusion criteria into our search (depicted in **Figure 1**), 328 articles closely related to the aims set forth for this review were selected and hence utilized.

### INCLUSION AND EXCLUSION CRITERIA

Articles on EE paradigms in rodents were mainly selected for detailed analysis. Articles which investigated the effects of an environmental stimulus on nervous and/or immune systems, but not specifically discussing EE were consulted in less depth and are cited wherever required for the convenience of readers. Similarly, analogous evidence to each EE method in human studies have also been included and cited. This raised the total number of articles cited to 328. EE paradigms on animals other than rodents were excluded due to their small numbers and to maintain uniformity. All articles included in this review have been published between 1988 and 2014. Articles without the full text available were excluded from the review.

## ROLE OF IMMUNE FACTORS IN NEUROBIOLOGY AND BEHAVIOR

Various cytokines and circulating T cells have been shown to play important role in hippocampal neurogenesis and in the molecular and cellular mechanisms responsible for learning, memory, and cognition under physiological conditions (McAfoose and Baune, 2009; Yirmiya and Goshen, 2011). Further, these immune factors also maintain homeostasis and influence molecular mechanisms involved in monoamine metabolism, the sensitivity of the hypothalamic–pituitary–adrenal (HPA) axis to cortisol and certain other cellular neuroimmune functions in constitutive levels (Eyre and Baune, 2012). Conversely, levels of both pro-inflammatory and anti-inflammatory cytokines in the peripheral circulation and CNS rise during several brain disorders such as depression, schizophrenia, and AD (Schwarz et al., 2001).

In the course of pathological conditions, such as stroke and related diseases (Kim, 1996) and environment adversities, such as social stress (Avitsur et al., 2005), a cytokine cascade is initiated and brain cells express various pro-inflammatory cytokines, chemokines, and adhesion molecules. The first two cytokines in the cytokine cascade, TNF-α and IL-1β (Th1 type, stimulatory), are pro-inflammatory and produced locally. They further activate granulocytes, monocytes/macrophages, NK cells, and T and B cells, and recruit them to the sites of inflammation (Petersen and Pedersen, 2005, 2006). Importantly, studies investigating the correlation between cytokine levels and occurrence of AD have reported the presence of cytokines TNF-α and IL-1β in cerebrospinal fluid (Tarkowski et al., 2003), and elevated plasma levels of cytokines IL-1β and IL-6 (Licastro et al., 2000) in patients, which suggests their active role in the pathophysiology of AD. Indeed, elevated levels of TNF-α in particular have been shown to cause a reduction in hippocampal volumes through the neurodegenerative TNF receptor 1 (TNFR1) pathway (Baune et al., 2012) and can lead to the development of depressive-like behavior (Eyre et al., 2013). A regression analysis on a cohort of non-demented community-dwelling adults aged between 70 and 90 years showed that increased levels of cytokines during systemic inflammation are related to cognitive deficit in a non-clinical community-dwelling population, independent of depression, cardiovascular and metabolic risk factors (Trollor et al., 2012), highlighting the significance of levels of cytokines in systemic circulation for brain function.

Experiments in rodents have revealed that the level of pro-inflammatory cytokines in the brain rise with aging and are directly related to age-related impairments in learning, memory, and cognition (Tha et al., 2000). This indicates that pro-inflammatory cytokines are involved in promoting neuroinflammation during old age and play a role in associated psychiatric disorders which are generally accompanied by memory and cognitive deficits. It should, however, be noted that pro-inflammatory cytokines can also stimulate anti-inflammatory pathways, through, for example, enhancement of the production of anti-inflammatory cytokines such as IL-1ra and IL-10. These anti-inflammatory cytokines can then inhibit the production of TNF-α and IL-1β (Opal and DePalo, 2000; Sredni-Kenigsbuch, 2002; Petersen and Pedersen, 2005), thereby reducing inflammation and marking the end of the cytokine cascade.

Several other humoral immune factors have also been reported to modify brain anatomy and functions. These include TLRs, mitogen-activated protein kinases (MAPKs), CRP, the complement system, chemokines, and immunoglobulins (Igs). The enhanced expression of TLR-3 and -4, the proteins expressed by glial cells and oligodendrocytes in the brain, has been observed in inflamed CNS tissues during immunohistochemical post-mortem brain analysis (Bsibsi et al., 2002). They are indeed reported to be actively involved in the modulation of innate (Medzhitov, 2001) and adaptive immune responses, and regulation of dendritic cell functions (Iwasaki and Medzhitov, 2004). Likewise, MAPKs which are specific protein kinases (serine–threonine specific), elicit pro-inflammatory and immunomodulatory functions (Lee et al., 1994; Dong et al., 2002) in the brain and their signaling is controlled by MKP-1 (MAPK phosphatase-1), a dual-specificity phosphatase. CRP, which is an acute phase reactant protein, has been shown to enhance inflammation and tissue damage by promoting phagocytosis by opsonization (Du Clos, 2000) and activating the complement system (Padilla et al., 2003). The latter consists of distinct plasma proteins that also act as opsonins and initiate a series of inflammatory responses (Janeway et al., 2001). Researchers have observed upregulation of the complement system in human brain during AD and other neurodegenerative diseases (McGeer and McGeer, 1995; Yasojima et al., 1999). Similarly, high levels of CRP in the brain have been linked to cognitive impairment and dementia (Kuo et al., 2005), and AD (McGeer et al., 2000). Chemokines are small cytokines that promote inflammation by attracting leucocytes to the point of inflammation and have also been reported to play a part in neuromodulation (Proost et al., 1996; Mélik-Parsadaniantz and Rostène, 2008). Contrary to all of the above factors, intravenous administration of IgG has been shown to induce anti-inflammatory action *in vivo* (Nimmerjahn and Ravetch, 2008) and could be beneficial in the treatment of AD (Dodel et al., 2004) by inhibiting the neurotoxic effects of amyloid-β (Aβ).

Although it was originally thought that the blood–brain barrier (BBB) provides an immune privileged status to the brain, RCTs in rodents have shown that freshly activated T cells migrate across the BBB during neuroinflammation, and along with macrophages/monocytes, are present at all times in the brain for immune surveillance (Hickey et al., 1991; Engelhardt, 2006). It is, however, important to note that T cells, particularly the Th1 and Th2 phenotypes, secrete various antagonistic cytokines (Th1 elicits pro-inflammatory response and Th2 elicits anti-inflammatory response) and thereby also control neuro-humoral immune responses during psychiatric disorders (Schwarz et al., 2001). The role of NK cells in various brain disorders such as depression, AD and PD has also recently been reviewed and validated by some researchers (Poli et al., 2013). While exchange of B cells across the BBB has been reported in patients with multiple sclerosis and associated with the development of autoimmunity in the CNS (von Büdingen et al., 2012), their role in psychiatric illnesses such as depression has not been studied in detail so far.

## ROLE OF GLIAL CELLS IN NEURO-IMMUNOMODULATION

Glial cells, microglia and astrocytes, are the primary immune effector cells and express various cytokines in the CNS (Rothwell et al., 1996; Hanisch, 2002). However, the source of cytokines in the brain can be central (via microglia and astrocytes), as well as peripheral (via monocytes, macrophages, Th17 cells, and other T cells) and certain cytokine signals reach the brain parenchyma through humoral, neural, and cellular pathways (see review by Capuron and Miller, 2011 for more information about these pathways).

Microglia are specialized macrophages and are considered the principal immune cells in the brain. They carry phenotypic markers for blood monocytes and tissue macrophages (McGeer et al., 1993) and are shown to be involved in immuno-surveillance and neuroprotection (Conde and Streit, 2006). In particular, microglia are known for the production of cytokines in the CNS and protecting it from numerous pathologies such as infectious diseases, trauma, ischemia, brain tumors, neuroinflammation, and neurodegeneration (Kreutzberg, 1996). A RCT on rodents has shown that microglia in association with cytotoxic T cells are important for neurogenesis, adult brain plasticity, and spatial memory (Ziv et al., 2006). Though microglia are neuroprotective, their overexpression or sustained stimulation can result in enhanced production of cytokines (e.g., IL-1β and TNF-α; Sawada et al., 1989; Hanisch, 2002), as well as in the expression of class I and II major histocompatibility complex antigens as seen in a RCT in rodents and in the post-mortem brain tissues of AD and age-matched control cases (Tooyama et al., 1990), respectively. This overexpression of microglia may lead to severe neuroinflammation, neurodegeneration, and subsequent cognitive dysfunction.

In the presence of an activating stimulus, microglia modulate the immune response by producing pro-inflammatory cytokines. This in turn recruits more microglia to the site, as well as attracts immune cells from the peripheral blood. Likewise, when the stimulus wanes, microglia participate in switching off of the immune response by producing anti-inflammatory cytokines that also causes their own apoptosis (Garden and Möller, 2006). Schwartz et al. (2006) suggested that activation of microglia into either of these forms is determined by the type of stimulus, its duration and its preceding, concomitant and subsequent stimuli.

The role and functions of microglia have been reviewed by many researchers in the past (Mrak and Griffin, 2005; Streit, 2005; Dilger and Johnson, 2008). These reviews report that microglia are primed with aging, become increasingly dysfunctional, lose their neuroprotective properties and upon secondary stimulation release excessive quantities of pro-inflammatory cytokines, such as TNF-α, IL-1β, and IL-6. This in association with genetic factors and acquired environmental risks, predisposes the brain to development of neurodegenerative disorders. Activated microglia and the released cytokines have also been reported to play a role in the formation of amyloid plaques and for the onset of neurodegeneration during aging that leads to AD (Licastro and Chiappelli, 2003; Streit, 2004). Interestingly, the activation of glial cells and expression of pro-inflammatory cytokines like IL-1α, IL-1β, and IL-6 with aging has been observed to be region specific in an *in vitro* model of primary glia cultured from brain regions of male Fisher 344 rats sampled across the life span, occurring more prominently in the hippocampus than in the cerebral cortex (Xie et al., 2003). This might cause neurodegenerative changes in brain regions like the hippocampus, with subsequent effects on cognition.

The discussion above, as well as some reviews (Liu and Hong, 2003; Glezer et al., 2007; Ekdahl et al., 2009), suggest that microglia can display both neuroprotective and neurotoxic effects depending on the extent of their cytokine expression, which therefore makes them the potential target for the treatment of neurological diseases.

Like microglia, astrocytes have neuroprotective functions in the normal brain but could be responsible for neurological diseases as well. They can repair damaged neural tissue, guide neuronal migration during development, mediate synaptic plasticity, act as antigen presenting cells and maintain the structural and functional integrity of the BBB (Montgomery, 1994). Similar to microglia, they are immune effector cells, expressing cytokines (IL-1, IL-6, IL-10, IFN-α and -β, TNF-α and -β) and chemokines, and mediating inflammation and immune reactivity in the brain. The under-expression or overexpression of astrocytes has been reported to cause neuroinflammation with resultant neurodegeneration (Dong and Benveniste, 2001), which emphasizes them as the second most important target in the brain, after microglia, for cytokine-modulation-based paradigms, such as EE.

## ENVIRONMENTAL ENRICHMENT

**FIGURE 2:**
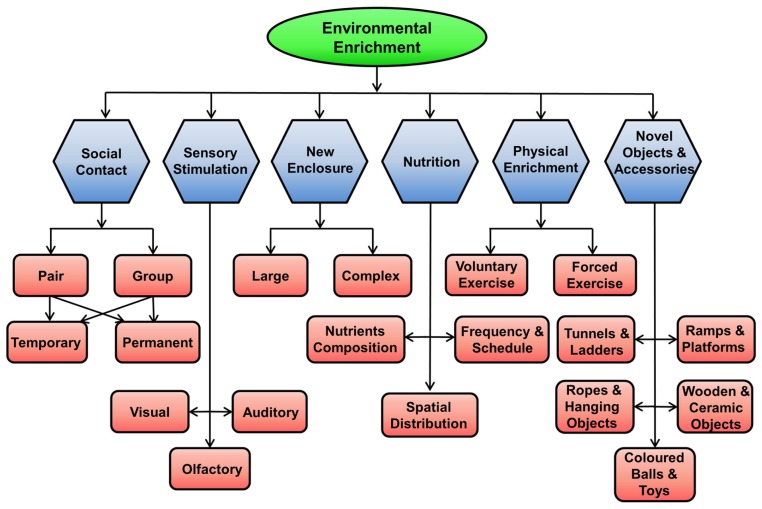
**Methods of enriching the environment for rodents in captivity.** EE for rodents in captivity can be achieved by providing them with social contact (pair, group, permanent, temporary), physical activity using running wheel, new larger and complex enclosures, novel objects and accessories, sensory stimulations (visual, auditory, and olfactory), and better nutrition.

The environment for rodents can be enriched in many ways, as shown in **Figure 2**.

### EFFECTS OF VARIOUS EE METHODS ON NEUROBIOLOGY AND BEHAVIOR

#### Physical exercise

***Rodent studies.*** This effectiveness of PE in enhancing neuroplasticity and improving brain function has been utilized in rodent studies for the purpose of enrichment of surroundings in several EE paradigms. RCTs on rodents have shown enhancement in hippocampal neurogenesis, improvement in cognition, learning, and memory performances with PE (Ahmadiasl et al., 2003; Van Praag et al., 2005; Nichol et al., 2009). Similarly, exercise on a running wheel promoted memory acquisition, memory retention, and reversal learning in rodents tested on a Y maze post-exercise treatment (Van der Borght et al., 2007). PE has also been shown to affect behavior in rodents. Indeed, long-term voluntary exercise has been shown to reduce anxiety- (Benaroya-Milshtein et al., 2004; Binder et al., 2004; Duman et al., 2008) and depressive-like behavior (Zheng et al., 2006; Duman et al., 2008; Marais et al., 2009).

Physical exercise on a running wheel when used in conjunction with toys and accessories (e.g., tunnels, ramps, bells, etc.) improved the tissue integrity and cognitive performances of rodents following severe traumatic brain injury (Hamm et al., 1996; Passineau et al., 2001). This combination has also been shown to induce a fivefold increase in hippocampal neurogenesis, enhancing learning, exploratory behavior and locomotor activity in aged mice (Kempermann et al., 2002). Enhanced spatial memory and an increase in dendritic arborization were seen in rats housed in groups and provided with a running wheel, shelter, and toys (Leggio et al., 2005), suggesting a possible beneficial effect of PE on dendritic morphology. In addition, PE has been shown to promote neurogenesis in the dentate gyrus of the hippocampus and modulate neural transmission across the synapses in the hippocampal region by modifying the extracellular concentration of neurotransmitters glutamate and gamma-aminobutyric acid (GABA) in the CA3 area of the hippocampus (Segovia et al., 2006). EE utilizing novel objects and accessories, with PE, ameliorated cognitive deficits in a mouse model of AD (Jankowsky et al., 2005), which could be due to the reduction of Aβ after enrichment (Lazarov et al., 2005). In another study, PE with social enrichment (five to eight mice in a cage) and novel objects and accessories improved spatial memory in mice (Jurgens and Johnson, 2012). The evidence above suggests a potential mechanism of PE, when used alone or in combination with other enrichment methods, in modulating neurobiology and behavior in rodents.

***Human studies.*** Recent reviews have upheld the long-standing view that aerobic exercise modulates neuroplasticity, and improves responsiveness to new challenges and psychosocial functions in humans (Hotting and Roder, 2013; Lees and Hopkins, 2013). In contrast, a recent meta-analytic study on random controlled trials of the effects of exercise on cognitive outcomes in adults aged over 65 years with mild cognitive impairment has rejected these claims stating that published results have very low statistical power and therefore are inconclusive (Gates et al., 2013). However, this may relate to the age of the subjects included within the analysis, as this limits the number and scope of articles for study. Moreover, the control cohort in the study included conditions like education, and stretching and aerobic exercise which may prove beneficial themselves, therefore preventing a positive finding for PE. Further, the intensity of exercise and external environmental conditions during PE can modulate its effects on brain (discussed later in this review), which were not considered in this review. Nonetheless these disparate findings show the complex nature of exercise physiology in human and animal interventions.

Physical exercise has been considered as an established and effective first-line treatment in mild to moderate depression. The role of PE in depression has been critically reviewed recently which suggests that the efficacy of PE in depression is classically attributed to its impact on changing certain neurobiological mechanisms including monoamine metabolism, HPA axis function, neurotrophic factors, neurogenesis, and neuroinflammation (Eyre and Baune, 2012). Like depression, individuals who were at risk of AD and dementia showed improved cognition after modest PE (Gleeson et al., 2011). Due to these beneficial effects of PE in psychiatric conditions, a suggestion that PE can be used as a non-pharmacological therapy for providing protection from neurodegenerative diseases, stress, and depression has been made by some authors in their reviews (Cotman et al., 2007; Hillman et al., 2008).

A RCT in human volunteers that underwent graded aerobic exercise training showed improvement in their spatial memory (Erickson et al., 2011), suggestive of a constructive role of moderate exercise in enhancing memory in humans. PE has also been shown to enhance cognition in healthy adult males after performing single acute bouts of moderately intense exercise, however, the authors observed that the effects of single acute bouts of moderately intense exercise improves only some aspects of cognition (primarily memory, reasoning, and planning) in healthy young individuals (Nanda et al., 2013). Nevertheless, a test of the effects of a single bout of moderate-intensity exercise on cognition may not be a reliable determination of its long-term effects (see review by Tomporowski, 2003). Interestingly, in a human intervention correlative study, it has been shown that these results can vary based on the pre-exercise performance of participants in the given task; post-exercise low performing participants perform better in the tests of cognition than high performing participants (Drollette et al., 2013). However, the authors of this study categorized preadolescent children into high performing and low performing children while resting, based on flanker task performance for incongruent trials. It is possible that high performing participants while resting showed performance relatively near to their peak levels, while performance of low-performing participants may have been low due to the lack of attention or other confounding variables. A similar paradigm in rodents where mice or rats are tested for cognition before and after exercise could be useful to accept the validity of results from this study.

***Summary of the role of physical exercise in EE studies.*** Overall, it is evident that the role of PE has been widely studied in both rodents and humans. It appears that PE modulates neurobiology and behavior via similar mechanisms in rodents and humans, particularly by enhancing the neuroplasticity of the dentate gyrus of the hippocampus, as seen in rodents (Segovia et al., 2006), and improves spatial memory, behavior, and cognition (Ahmadiasl et al., 2003; Van Praag et al., 2005; Duman et al., 2008; Nichol et al., 2009; Erickson et al., 2011; Nanda et al., 2013). PE is also known to enhance the levels of neurotrophins (Griffin et al., 2009) and modify the extracellular concentration of neurotransmitters (Lazarov et al., 2005) in the hippocampus, in addition to modulating neuroimmune mechanisms (Eyre and Baune, 2012; Speisman et al., 2013; Eyre et al., 2013) which are discussed later in this review. Moreover, the effects of PE on the brain are dependent on its duration and intensity (Mathur and Pedersen, 2008).

#### Social and cage enrichment

***Rodent studies.*** Profound effects on the behavior of captive animals when housed in groups could influence the methodological framework and validity of results in EE studies. A study suggests that male mice prefer sleeping in close proximity to a familiar mouse (Van Loo et al., 2004), which represents their preference for social environment. On the other hand, mice that were devoid of social contact showed signs of increased anxiety and depressive-like behavior with greater tendencies to attack other mice (Ma et al., 2011).

Further research has shown the evolution of two kinds of populations in laboratory rodents within a socially enriched environment: “dominant” and “subordinate,” both with different physiological and behavioral profiles (Bartolomucci et al., 2001; Cacho et al., 2003). While this has been shown to improve the performance of dominant animals, submissive or socially low ranked animals can suffer from social stress, may show immunosuppression and are usually more susceptible to viral infections and formation of tumors (de Groot et al., 1999; Azpiroz et al., 2003). The adverse effects of social stress on neuronal structure and neurochemical transmission (Blanchard et al., 2001), as well as on the morphology of hippocampal neurons, which are vital for learning and memory (Buwalda et al., 2005) has been suggested, indicating that social stress in a socially enriched environment could alter the findings of investigation on brain function. Indeed, from other studies, it appears that these two populations also exhibit distinct differences in anxiety-like behavior, with dominant mice showing anxiolytic behavior after repeated victory (Haller and Halasz, 2000), while subordinate mice showing anxiogenic-like and decreased exploratory behavior (Keeney and Hogg, 1999). The development of this hierarchy in the population of rodents poses a serious threat to EE paradigms which are based on the principles of enhancement and not impoverishment of the external environment.

These adverse behavioral and physiological changes due to social defeat (i.e., losing a confrontation among animals of same species) can be long lasting. However, mice that were housed together after social defeat showed improvement in behavior (Ruis et al., 1999). Similarly, a study in rats revealed that social housing after social defeat reverses the reduction in heart rate, temperature and locomotor activity caused by social defeat (de Jong et al., 2005). It appears that while social conflict may be harmful, social enrichment in the absence of these conflicts (e.g., when submissive rodents are housed together) is beneficial for neurobiology and behavior.

Findings from RCTs on rodents suggest that access to nesting material and nest boxes, increased complexity of the environment in cages and bedding material consisting of large particles (e.g., wood shavings) and fibers (e.g., shredded paper) are the natural preferences of rodents (Blom et al., 1996; Van de Weerd et al., 1998a,b; Olsson and Dahlborn, 2002). However, access to the preferred material can incite the expression of territorial behavior, for example, aggression, in rodents. This hypothesis was confirmed when mice provided with a bigger and more complex cage and more objects to explore were more aggressive, but interestingly this aggression was reduced when they were given access to nesting material (Van Loo et al., 2002). A plausible explanation for this could be that nesting material diverts the attention of rodents in cages from their conspecifics. Similar to this, another RCT showed that bedding material can partly be used to compensate for the deprivation of social contact (Van Loo et al., 2004). Notably, Fano et al. (2001) conducted an experiment to investigate agonistic behavior in male mice and reported that these paradigms of aggression are dependent on the intensity and duration of agonistic behavior and the interaction experience accumulated.

***Human studies.*** It is difficult to compare social and cage enrichment in rodents to humans. In humans, a possible equivalent could be active social behavior. An excellent review on human social behavior details the social psychology of humans and associated neurobiology (Adolphs, 2003).

***Summary of the role of social enrichment in EE studies.*** All of the findings above suggest that a subtle balance between social and cage enrichment is vital during EE studies in rodents. This could be achieved through meticulous planning while designing EE models. Limited research has been conducted on the neuromodulatory effects of social and cage enrichment methods on rodents at this stage.

Social enrichment in humans is a more complicated phenomenon and comprises a myriad of disciplines, such as social neuroscience, cognitive science, sociobiology, evolutionary psychology, and social psychology, converging together. Given these complex interactions, it is outside the scope of this review. It should be noted, however, that rodent models of social enrichment may not be ideal for investigating the effects of enrichment with social environmental stimuli in humans.

#### Enrichment with novel objects and accessories

***Rodent studies.*** The environment of rodents can be enriched with novel objects, puzzles (mazes, plastic tubes in different configurations) and accessories (toys, ropes, ladders, tunnels, hanging objects, house, ramps, and platforms) to stimulate their attention and engagement in the environment. Mice given access to novel objects and accessories with or without PE exhibited higher visuo-spatial attention and improved spatial memory when tested on the Morris water maze test (Tees, 1999; Williams et al., 2001; Bennett et al., 2006; Harati et al., 2011), which could be a function of increased hippocampal integrity and levels of neurotrophins in the hippocampus (Pham et al., 1999b; Gobbo and O’Mara, 2004). The reduction in cytochrome *c* oxidase levels in brain regions such as the infralimbic cortex, the paraventricular thalamic and hypothalamic nucleus, the basolateral amygdala, and the ventral hippocampus (Sampedro-Piquero et al., 2013) after enrichment with various objects and accessories could be another possible mechanism for this effect.

Enrichment in large cages furnished with various toys and accessories has been shown to restore the age-related loss of synaptophysin in aged mice. However, the authors observed no change in the number of synapses after enrichment, suggesting that enrichment improves synaptic plasticity by strengthening the synapses, not by formation of new synapses (Nakamura et al., 1999). An increase in play behavior, aggression and locomotor activity was observed in studies primarily investigating immunological alterations in mice after enriching them in complex cages furnished with a variety of items (Marashi et al., 2003, 2004). This method of enrichment has primarily been used in combination with PE, with further studies needed to investigate the effects of stimulation with novel objects alone.

***Human studies.*** Similar experimental paradigms in humans, i.e., activities that can stimulate attention and engagement in the environment, involve the provision of cognitively stimulating lessons (e.g., connecting dots to make an umbrella, naming easily identifiable objects after showing their pictures; Breuil et al., 1994), cognitive training (e.g., verbal episodic memory, reasoning, and visual search and identification; Willis et al., 2006), and cognitive rehabilitation (Clare et al., 2003). Studies on cognitive stimulation reported improvement in the tests of cognition (Breuil et al., 1994) and quality of life (Spector et al., 2003) by the participants. Likewise, improvement in memory and cognition of healthy volunteers, patients in the early stages of AD and vascular dementia, as well as major depressive disorder patients after cognitive training (Willis et al., 2006), cognitive rehabilitation (Clare et al., 2003), and cognitive remediation (Bowie et al., 2013), respectively suggests that these treatments can affect cognition in the healthy brain as well as in neuropathological conditions. These findings of the effects of cognitively enriched environment on cognition are further validated by meta-analytic studies on cognitive remediation for schizophrenic patients (McGurk et al., 2007; Wykes et al., 2011). Although these treatments, different from the method of providing EE with novel objects and accessories in rodents, they do promote a similar improvement in memory and cognition.

***Summary of the role of enrichment with novel objects and accessories in EE studies.*** This is the most common method of EE in rodents, after PE, and has been used extensively either as a stand-alone treatment or in conjunction with PE and social enrichment. EE with novel objects and accessories enhances memory and cognitive functions in rodents. Similar effects on cognition have been reported in humans in response to cognitive stimulation activities. The biggest question here is whether these treatments in rodents and humans are mechanistically similar. The perception of novelty for rodents in existing EE studies is “anything new” that rodents have not been exposed to so far. The same may not be applicable to humans. Novelty detection in humans has been shown to be a function of hippocampal (Knight, 1996) and amygdala (Blackford et al., 2010) activity and could depend on the participants within the study. This indicates that formulating a protocol with novel objects and activities according to the interests and likes of human participants, for example, sports equipment, magazines, or movies could be more appropriate method of translating EE paradigms in rodents to human studies.

#### Sensory enrichment

***Rodent studies.*** The effects of sensory enrichment on the activity of sensory organs (visual, auditory, and olfactory) and brain functions such as cognition and behavior in various captive animals have been reviewed and validated by Wells (2009).

*Visual stimulation.* Enrichment with objects that stimulate the visual cortex such as toys of different color and sizes, leafy plants, tree branches, and scattered food (to explore) has been shown to increase the thickness, number, and length of neurons, dendritic complexity, and spine density of the occipital cortex and improve visual processing activity in rodents (Venable et al., 1989; Piche et al., 2004; Rasin et al., 2011). This could be due to the enhanced levels of neurotrophins as observed in the visual cortex of newborn rats on exposure to light (Castren et al., 1992). Indeed, studies have shown that neurotrophins increase the length and complexity of dendrites (McAllister et al., 1995), potentiate excitatory synaptic transmission (Carmignoto et al., 1997) and enhance long-term potentiation in the visual cortex of rodents (Akaneya et al., 1997). Conversely, studies that investigated the effects of different light intensity and colors on visual stimulation suggested that mirrors (Sherwin, 2004) and some colors such as red (Sherwin and Glen, 2003) can be aversive and may affect emotionality and performance of mice.

*Auditory stimulation.* RCTs on rodents have shown that pure tone bursts (of different frequencies and intensities) and/or different tones (from hanging chains, wind chimes and bells) enhanced neuroplasticity, number of neurons, basal dendritic length, and spine density in the auditory cortex. This in turn improved directional sensitivity and increased response strength, threshold, selectivity, latency of auditory cortical neurons, and reorganization in the processing of spectral and temporal input in the posterior auditory field (Dinse, 2004; Engineer et al., 2004; Cai et al., 2009; Zhang et al., 2009; Bose et al., 2010; Jakkamsetti et al., 2012). According to some authors, auditory experience during early life can define the functional organization of the auditory cortex and enhance its processing capabilities to discriminate various auditory stimuli (Zhang et al., 2001; Xu et al., 2009).

In studies where adult mice were exposed to music with a slow rhythm, the authors observed enhanced learning performance and higher brain-derived neurotrophic factor (BDNF) levels in the hippocampus (Angelucci et al., 2007a) and hypothalamus (Angelucci et al., 2007b) of mice. The modulating effect of music on BDNF signaling has also been seen in the brain of mice exposed to Mozart’s piano sonata for approximately 7 days while in uterus and 60 days postpartum. However, these authors observed a decrease in BDNF levels in the auditory cortex though it increased in the cerebellum (Chikahisa et al., 2006). These results were unexpected, with findings from other studies suggesting that the neurotrophins NT-3 and BDNF can prevent the loss of auditory neurons (Staecker et al., 1996) and that BDNF signaling is important for shaping off of the experience-dependent plasticity in the auditory cortex during early postnatal life (Anomal et al., 2013). However, the authors only tested one type of music and the influence of the mother on the pups was not considered. This gives rise to the possibility that music like Mozart’s piano sonata might not be a favorable type of music to utilize in mice and the presence of the mother during music sessions could have affected the development of auditory acuity in new born pups. A hypothesis that different kinds of music induce distinct change in brain functions in rodents could therefore be explored.

*Olfactory stimulation.* A relation between olfactory stimulation and the brain is well documented, and is supported by the findings that olfactory bulbectomized rats show depression-like behavior (Kelly et al., 1997). Different odors stimulate the olfactory bulb which directly communicates with the olfactory cortex, hippocampus, amygdala, and hypothalamus in the brain and can induce behavioral changes. Enriched olfactory experiences in early life have been shown to enhance the functions of the adult olfactory bulb (Rabin, 1988; Rosselli-Austin and Williams, 1990), while odor deprivation in neonates reduced neurogenesis and the survival of the neurons (Corotto et al., 1994), as well as increased apoptosis in the olfactory bulbs of adult rodents (Najbauer and Leon, 1995). A study using an experimental paradigm that investigated the effects of enriched odor on 2-month-old mouse brains reported no effect on hippocampal neurogenesis or spatial memory on exposure to enriched odors; but an increase in the number of neurons in the olfactory bulb and improvement in odor memory were seen (Rochefort et al., 2002). While this study is suggestive of no effects of enriched odors on cognition and memory, it is unlikely that stressful odors such as smell of rotten food or injuries will also have no effect on behavior, memory, and cognition. Indeed, avoidance response has been shown in healthy rats to sickness-related odor cues (Arakawa et al., 2010). Moreover, novelty in odor determines the extent of improvement in short-term odor memory and neurogenesis in the olfactory bulb, which has been shown to be mediated by nor-adrenergic mechanisms (Veyrac et al., 2008). Further investigation on olfactory stimuli could clarify the significance of different odors in EE studies.

#### Human studies

*Visual stimulation.* Depending on the nature of external visual stimuli (favorable or aversive), contrasting neurobiological and behavioral outcomes are plausible. Distinct arousal of emotions and enhanced episodic recognition memory have been observed in response to pleasant and aversive visual stimuli, and found to be related to amygdala activity (Hamann et al., 1999). The role of different visual stimuli in evoking emotions and behavior in day-to-day human life is evident. However, a study in humans has shown that 1 min of exposure to blue light can trigger stimulation of cognitive brain activity in visually blind individuals (Vandewalle et al., 2013), suggesting that photoreception can modulate brain functions even in the absence of image formation. On the other hand, other sensory modalities such as tactile tasks could also activate the visual cortex, as seen in response to braille reading in blind subjects (Sadato et al., 1996). Indeed, activities like cognitive training involving visual tasks (e.g., recalling pictures after seeing them briefly; Breuil et al., 1994) could also provide visual stimulation to participants.

*Auditory stimulation.* While daily activities involve listening to various sounds, auditory enrichment for humans primarily involves listening to music of your own choice. Beneficial effects of music on the well-being, mood, learning performance, and cognitive development in humans are well known (McCraty et al., 1998; Kemper and Danhauer, 2005; Rickard et al., 2005; Hars et al., 2014). Music therapy has been shown to reduce irritability and depression (Hanser and Thompson, 1994; Ragneskog et al., 1996; Maratos et al., 2008), as well as improve emotional and behavioral responses in dementia patients (Sherratt et al., 2004), suggestive of its significance for the treatment of psychiatric conditions. Indeed, music could even be more potent in reducing depression than psychotherapy (Castillo-Pérez et al., 2010). Music has also been shown to enhance cognitive ability. In an experiment on patients with a left or right hemisphere middle cerebral artery stroke, listening to self-selected music for 2 months improved mood and enhanced cognitive recovery (Särkämö et al., 2008). Some authors have proposed that music therapy can be used as an alternate therapy in psychiatric conditions like depression and schizophrenia (Lin et al., 2011). Moreover, a review suggested that learning mechanisms mitigating effects of auditory stimuli on the brain could be applied to better understand the biology underlying everyday learning (Strait and Kraus, 2014). However, it is possible that different kinds of music could correlate to differential effects on the brain of psychiatric patients, as seen in healthy volunteers (Möckel et al., 1994; Blood et al., 1999).

*Olfactory stimulation.* Olfactory stimulation with a pleasant odor could improve learning and behavior in humans and indeed has a role to play from the first week after birth. A review by Schaal (1988) suggests that olfactory cues activate the olfactory bulb and help infants in the first postnatal week to bond with their mother and differentiate familiar from unfamiliar individuals. In his review, Herz (2009) analyzed the effects of various odors on behavior in humans and suggested that certain odors such as that of sandalwood or those self-selected by participants as pleasant could be used for the treatment of anxiety, depression, and insomnia. It has been shown that olfactory deficits may predict AD or PD in the patient (Mesholam et al., 1998; Devanand et al., 2000). Indeed, neurodegenerative and psychiatric diseases have been shown to reduce olfactory bulb neurogenesis in humans (Turetsky et al., 2000; Winner et al., 2011). Moreover, an aversive olfactory stimuli, for example, odor of a mixture of sulfide gasses, could even initiate emotions by activating the amygdala (Zald and Pardo, 1997). These findings clearly suggest that olfactory cues might have a role to play in psychiatric conditions.

***Summary of the role of sensory enrichment in EE studies.*** Taken together, studies on sensory enrichment have shown prominent effects on the neurobiology and behavior of rodents, with analogous evidence also evident within human literature. A similarity between sensory enrichment and enrichment with novel objects and accessories in rodent studies can be seen, since the standard environment of rodents in captivity is devoid of any special object or sensation. As such, a new sensation can be a novel input for effecting changes to brain function, such as cognition, in rodents. It is likely that the sights of aversive stimuli such as that of dominant or injured animals and favorable stimuli such as the introduction of a running wheel or novel toys could influence behavior and cognition of rodents in itself. The opposite, that enriched environments can enhance visual processing activity is also possible, as suggested by Cancedda et al. (2004). A possible explanation for this could be enhancement of the levels of neurotrophins in the visual cortex which promote neurogenesis, when environment is enriched with running wheels, and novel objects, toys and accessories (Torasdotter et al., 1998; Pham et al., 1999a).

See **Table 1** for studies detailing effects of different enrichment methods on neurobiology and behavior.

**Table 1:** Effects of environmental enrichment on neurobiology and behavior.

Study’s primary objective	Animal species/strain	EE methods	Frequency of changing EE method	Protein/behavioral parameters investigated	Significant findings	Reference
• Effects of EE on neurodegeneration during AD	• AD11 mice	• Large cages with wire mesh lid, several food hoppers, running wheel and objects of different shapes (tunnels, shelters, stairs, boxes)	• Once per week	• Visual object recognition test	• ↑ Visual object recognition memory and spatial memory	Berardi et al. (2007)
				• Morris water maze test	• ↓ Aβ deposition in hippocampus
				• IHC	• ↓ Progression of neurodegeneration
• Effects of long-term EE on hippocampal neurogenesis	• 10 months old female C57BL/6 mice	• Large cages, with re-arrangeable set of plastic tubes, a running wheel, nesting material, and toys	–	• Behavioral testing with Activity chamber, Rotarod, and Water maze	• Fivefold increase in hippocampal neurogenesis in enriched environment	Kempermann et al. (2002)
			• IHC and IF for lipofuscin deposits in neurons	• ↑ Learning, exploratory behavior, and locomotor activity
				• ↓ Lipofuscin deposits in the dentate gyrus
• Effects of environmental complexity on spatial abilities, dendritic arborization, and spine density	• Wistar rat (21 days old)	• Ten rats in a large cage of two levels connected by ramps, containing wood shavings, a running wheel, a shelter, plastic colored toys, and constructions	• Once a week	• Full-baited maze procedure and forced-choice procedure, performed in a radial maze	• ↑ Performance in the Radial maze and Morris water maze tasks	Leggio et al. (2005)
			• Morris water maze	• ↑ Dendritic arborization and spine density in layer-III parietal pyramidal neurons	
			• *In vivo* Golgi-like filling of the neurons for the visualization of dendritic arborization		
• Influence of EE on neurotrophins levels in the cerebellum	• Adult male Wistar rats	• 10 rats in a large cage with two levels, connected by ramps, contain wood shavings, a running wheel, a shelter, plastic colored toys, and constructions	• Twice per week	• Determination of BDNF and NGF in all brain regions using ELISA	• ↑ BDNF levels in the cerebellum, frontal cortex and hippocampus	Angelucci et al. (2009)
					• ↓ BDNF levels in the striatum
					• ↑ NGF levels in the cerebellum and striatum
					• No significant change in NGF levels in the frontal cortex and hippocampus
• Effects of EE on the neurogenesis and the extracellular concentrations of glutamate and GABA in the hippocampus	• Male Wistar rats of 2 and 25 months	• Two running wheels, a re-arrangeable set of plastic tunnels, an elevated platform, toys	• Every 3–4 days	• Water maze test	• ↑ Spatial memory performance	Segovia et al. (2006)
				• Neurogenesis in the dentate gyrus of hippocampus using BrdU labeling	• ↑ Hippocampal neurogenesis in both young and aged enriched rats
				• Glutamate and GABA concentration in CA3 region of hippocampus using microdialysis probes	• ↓ Hippocampal neurogenesis in control rats
					• No effect of EE on basal concentration of Glutamate and GABA in young rats
					• ↑ Basal glutamate and GABA concentration in old rats
• Effects of ageing and EE on synaptic plasticity	• Male Fischer rats	• Large cages furnished with various toys and small constructions	–	• Electron microscopic morphometry for the analyses of density and sizes of synapses	• ↓ Synaptic vesicle density with age	Nakamura et al. (1999)
					• EE restored age-related loss of synaptophysin

*EE, environmental enrichment; AD, Alzheimer’s disease; IHC, immunohistochemistry; Aβ, amyloid-β; IF, immunofluorescence; BDNF, rain-derived neurotrophic factor; NGF, nerve growth factor; ELISA, enzyme linked immunosorbent assay; BrdU, bromodeoxyuridine; GABA, gamma-amino butyric acid; CA, Cornu Ammonis.*

### THE INFLUENCE OF EE ON NEUROBIOLOGY AND BEHAVIOR VIA MODULATION OF CYTOKINES AND IMMUNE CELLS

#### Physical exercise

***Anti-inflammatory and humoral immune mechanisms of physical exercise.*** Several studies have reported the anti-inflammatory effects of PE during diseases and metabolic disorders which are associated with chronic low-grade systemic inflammation such as cardiovascular disease and type II diabetes mellitus (Petersen and Pedersen, 2005; Wilund, 2007). PE has also been shown to slow down cellular aging which is generally associated with inflammatory conditions, an increased occurrence of circulating autoantibodies and lymphoproliferative disorders and hence greater morbidity and mortality rates (Shinkai et al., 1998; Senchina and Kohut, 2007).

Several mechanisms have been investigated and cited for the anti-inflammatory effects of PE. It is clear that PE affects skeletal muscles which are able to act as an endocrine organ in body as they release myokines/cytokines on contraction thereby influencing metabolism and modifying cytokine production in other tissues and organs (Petersen and Pedersen, 2006). However, PE also increases the secretion of cortisol and adrenaline from the adrenal glands, enhances the production and release of IL-6 and other myokines from working skeletal muscles and reduces the expression of TLRs on monocytes and macrophages. Research suggests that this increase in IL-6 in response to PE is dependent on its intensity, duration, the mass of muscle recruited, and endurance capacity (Petersen and Pedersen, 2005; Mathur and Pedersen, 2008).

The production and release of IL-6 from muscle fibers is important as it enhances lipid turnover by stimulating lipolysis as well as fat oxidation, thereby reducing the production of adipokines including TNF-α, leptin, retinal-binding protein 4, lipocalin 2, IL-6, IL-18, CCL2, and CXCL5 (Petersen and Pedersen, 2005; Eyre and Baune, 2012). Evidently the increase in energy expenditure associated with exercise also assists in promoting lipolysis and reducing production of adipokines (Gleeson et al., 2011). Moreover, IL-10 produced in response to IL-6 acts as an anti-inflammatory molecule and further inhibits the production of IL-1α, IL-1β, and TNF-α as well as the production of chemokines. Additionally, another anti-inflammatory mechanism of PE, where it inhibits monocyte and macrophage infiltration into adipose tissues as well as stimulates phenotype switching within adipose tissue has also been suggested (Eyre and Baune, 2012).

Physical exercise is also likely to suppress TNF-α via IL-6-independent pathways, since a modest decrease of TNF-α after PE was still seen in IL-6 knockout mice (Keller et al., 2004). High levels of cortisol and epinephrine are triggered by PE due to the activation of the HPA axis and the sympathetic nervous system, and this cortisol and epinephrine infusion in turn has been shown to blunt the appearance of TNF-α in response to endotoxin *in vivo* (Petersen and Pedersen, 2005; Gleeson et al., 2011). In a study on resting subjects, endotoxin induced a two- to threefold increase in circulating levels of TNF-α. In contrast, when the subjects performed 3 h of ergometer cycling and received the endotoxin bolus at 2.5 h, the TNF-α response was completely diminished (Petersen and Pedersen, 2005). However, the mechanism whereby cortisol and epinephrine inhibit TNF-αproduction is still not clear. It appears that epinephrine and IL-6 inhibit endotoxin-induced production of TNF-α via independent mechanisms. The possibility exists that, with regular PE, the anti-inflammatory effects of an acute bout of PE will protect against chronic systemic low-grade inflammation, but such a link between the acute effects of PE and the long-term benefits has not yet been proven.

***Cellular immune mechanisms of physical exercise.*** Changes to cellular immunity in response to PE have also been reported by several authors. Leukocytosis is commonly seen during exercise, the extent of which is related to the intensity and duration of exercise. However, the cellular changes post-exercise are determined mainly by the time elapsed since starting exercise and not the work intensity and the total work done (McCarthy and Dale, 1988). A study on eight internationally competitive oarsmen, undergoing 6 min of “all-out” bouts of ergometer rowing over 2 days showed that compared with levels at rest, the first bout of exercise increased the concentration of leukocytes (twofold); neutrophilic granulocytes (twofold); lymphocytes (twofold); monocytes (twofold); the blood mononuclear cell (BMNC) subsets CD3^+^ (twofold), CD4^+^ (twofold), CD8^+^ (threefold), CDl6^+^ (eightfold), CDl9^+^ (twofold), and CDl4^+^ (twofold); the NK cell activity (twofold); and plasma IL-6 (threefold). The increase in leukocytes, neutrophilic granulocytes, lymphocytes, the BMNC subsets CD4^+^, CD8^+^, CD16^+^, CD19^+^, and CD 14^+^, as well as in the NK cell activity was even higher after the last bout of ergometer rowing by one- to fivefold. More importantly, all above values were at or more than the levels at rest during the recovery period. Indeed, leukocytosis, neutrophilocytosis, lymphocytosis, and higher NK cell activity was observed even on the day after the bout. This study is a good example of how PE can modulate levels of immune cells in the blood and improve cellular immunity (Nielsen et al., 1996). Significant cytological changes post-exercise have also been observed by Nehlsen-Cannarella (1998). The authors observed that immediately after PE, there was an increase in both the circulating leukocyte and neutrophil count, but only a small increase in the monocyte count. This was followed by a further increase in neutrophil numbers, although leukocyte numbers fell below the pre-exercise levels. Accompanying the increase in neutrophil count was the marked release of pro-inflammatory cytokines (TNF-α, IL-6, and IL-1) followed by IL-1 receptor antagonists, the products of monocytes and tissue macrophages, as well as brain glial cells. Moreover, while the number of NK cells show increase after moderate exercise, they could decline after high-intensity PE (Jankowsky et al., 2005). Further, their activity on a per cell basis remains the same (Duman et al., 2008; Marais et al., 2009) or increases (Griffin et al., 2009), depending on the intensity and duration of exercise. In a study where healthy volunteers underwent 60 min of bicycle exercise at 75% maximal oxygen uptake (Vo_2max_), decline in T helper cells (CD4^+^ cells) and increase in NK cell subset (CD16^+^) were seen in the blood (Tvede et al., 1989), consistent with the other findings above. Moderate PE has also been shown to affect various functions of neutrophils such as enhancing phagocytosis (Ortega et al., 1993) and production of microbicidal reactive oxygen species (Smith et al., 1990) by neutrophils. Both short-term and chronic PE improve the function of macrophages by enhancing phagocytosis, however, exhaustive PE has been shown to suppress antigen processing by macrophages (Ceddia and Woods, 1999; Ortega et al., 2007). This suggests that moderate exercise could be useful for inducing beneficial immune changes in the body, while exhaustive exercise could be harmful to the immune system.

***Physical exercise modulates cytokines and other humoral and cellular immune factors in the brain.*** Systemic cytokines can cross the BBB and affect various brain regions including the hippocampus, cerebellum, pituitary, and cortex. This has been reviewed in detail by several authors (Banks et al., 1995; Banks and Erickson, 2010), which suggests that any change in the systemic cytokine levels during infections can potentially affect brain function. However, this association of brain function with systemic cytokine levels requires further investigation. In addition, cytokines produced and expressed within the brain can also initiate neuroimmune reactions on their own. For instance, in the study conducted by Tarkowski et al. (2003), the authors observed low levels of TNF-α in the serum compared to the cerebrospinal fluid which provided evidence for local production of TNF-α within the brain rather than in the periphery. The observed presence of high levels of TNF-α in the brain of dementia patients indicates active neuroinflammation with resultant neurodegeneration, which contributes to the pathophysiology of several brain diseases.

Higher levels of complement component C4 and CRP has been observed in the serum of patients with major depression (Berk et al., 1997). Similarly, mRNAs of all components of the classical complement pathway are increased, particularly C1q mRNA by 11- to 80-fold and C9 mRNA by 10- to 27-fold over control levels in the entorhinal cortex, hippocampus, and midtemporal gyrus regions of the brain in patients with AD (Yasojima et al., 1999). Activated complement components have also been observed in the affected brain regions of patients with PD (McGeer and McGeer, 2004) and age-related macular degeneration (Anderson et al., 2010). These results further confirm that neuroinflammatory and neurodegenerative processes drive the pathophysiology in depression, AD, PD, and other aging-related brain diseases (McGeer and McGeer, 2003; McGeer et al., 2005).

Physical exercise has been shown to reduce inflammation and oxidative stress in the brain. A study by Speisman et al. (2013) in aged rats showed that daily voluntary exercise on a running wheel decreased hippocampal IL-1β and serum monocyte chemoattractant protein-1 (a chemokine that regulates migration and infiltration of monocytes/macrophages from the blood across the vascular endothelium, a key mechanism during inflammation). However, rather surprisingly, the authors also observed increased IL-18 concentration in the hippocampus, which has pro-inflammatory functions. Since, levels of IL-18 correlated with hippocampal neurogenesis, the authors suggested that the pro-angiogenic properties of IL-18 might have improved vascular health and hence stimulated hippocampal neurogenesis. Several other immune pathways have also been proposed for the anti-neuroinflammatory effect of PE (Eyre and Baune, 2012; Eyre et al., 2013). These include (i) increased attraction of macrophages into the CNS and hence enhancement of their regulatory effects on neurotoxic microglia, (ii) upregulation of MKP-1 which plays an essential role in negatively regulating the pro-inflammatory macrophage MAPK activation, and (iii) modulation of hippocampal T cells which are responsible for neuroregeneration and for modulation of microglia. Moreover, certain types of exercise could have greater effects on the immune factors than others and modulate anti-inflammatory mechanisms by influencing several immune factors at the same time. For instance, in a RCT on older adults, aerobic exercise treatment resulted in significant reductions in serum CRP, TNF-α, IL-6, and IL-18 in the participants while flexibility/resistance exercise only caused a decrease in serum TNF-α levels (Kohut et al., 2006). Further, the possible anti-inflammatory and immunomodulatory effects of change in the levels of systemic cytokines and immune cells after PE on the brain cannot be overlooked, although this needs further analysis.

***Summary of the neuro-immunomodulatory role of PE in EE studies.*** In terms of the neuroimmune effects of PE, a similarity is seen in human and rodent studies. Exercise has been shown to reduce levels of TNF-α and IL-1β, as well as certain cellular biomarkers in the brains of rodents and humans. However, human and rodent studies assessing the positive effects of PE on neuroimmune mechanisms are difficult to compare due to the utilization of different types, durations and intensities of the exercise and inconsistencies in the immune markers investigated (Eyre and Baune, 2012). Although, PE can be used either as a stand-alone or adjunctive therapy, and has preventative properties for brain pathologies, monitoring and controlling the external environmental variables could be very important to achieve the desired effects on neuroimmune mechanisms.

It has been shown that frequent bouts of PE with exposure to harsh environments such as extremes of heat, cold and humidity, as well as pathogens and stressors to the immune system including lack of sleep, severe mental stress, malnutrition, and bodyweight loss can precipitate diseases associated with inflammatory conditions (Neiman and Pedersen, 1999), which in many respects mimics the immune reactions observed in clinical sepsis (Shephard and Shek, 1998). This indicates that exhaustive exercise or acute bouts of exercise in adverse environmental conditions may act as a deterrent to the normal functioning of the immune system, inducing immunosuppression and increased susceptibility to infections. Some authors have also reported a correlation between PE, external environment temperature and immune changes in the body. Brenner et al. (1999) observed greater elevation in the number of immune cells, such as leucocytes, neutrophils, and NK cells after PE in a hot environment. Prior exercise has been shown to significantly augment leukocyte, granulocyte, and monocyte response to cold exposure (Brenner et al., 1999). This suggests an association between PE, external environmental conditions, and immunological changes in the body and indicates that several external environmental variables can interfere with the results in exercise-based paradigms.

**FIGURE 3:**
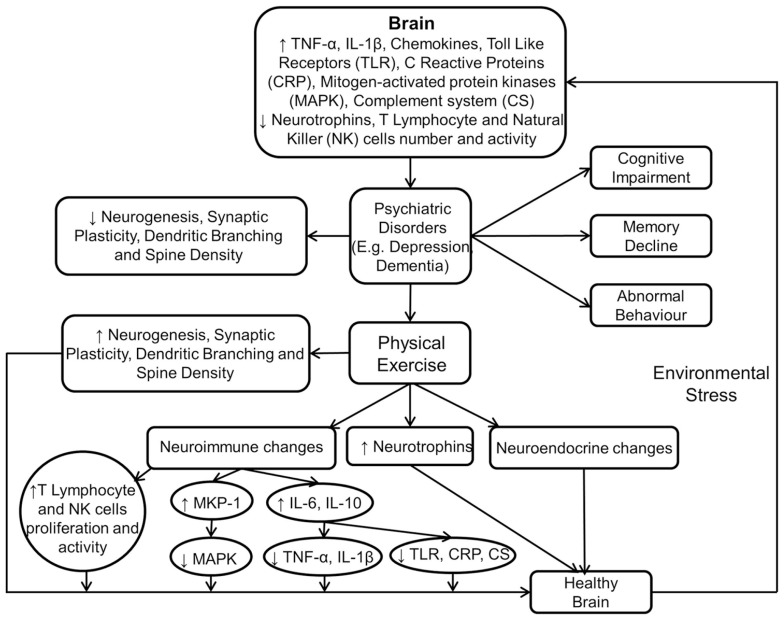
**Physical exercise modulates neuroimmune and neuroendocrine mechanisms to induce beneficial effects in the brain.** This figure shows that psychiatric disorders are the result of aversive neurobiological and neuroimmune changes in the brain. However, these are reversed by physical exercise which also facilitates improvement in cognition, memory, and behavior.

**Figure 3** shows the immunomodulatory mechanism of physical exercise in producing beneficial neurobiological and behavioural effects.

#### Other forms of environmental enrichment

***Social enrichment.*** Noticeable alterations in the humoral and cellular immune responses have been observed in animals reared in a socially enriched environment, particularly in submissive animals. These include reduction in the proliferation of splenocytes, and the production of some cytokines (IL-4 and IL-10) and serum antibodies in subordinated animals (Fleshner et al., 1989; Bartolomucci et al., 2001). Similarly, a decrease in T cell proliferation and IL-2 production in submissive animals (Hardy et al., 1990) and a reduction in the number and activity of T cells and NK cells (Stefanski and Engler, 1999; Stefanski, 2001) have been reported after severe social stress. In another experiment, 2 h of social confrontation led to an increase in the number of granulocytes, a decrease in lymphocyte numbers and elevated CD4/CD8 and T cells/B cells ratio in defeated animals (Stefanski and Engler, 1998). These findings clearly suggest that immunosuppression in subordinate animals is caused by the impairment of both humoral and cellular immunity, possibly from social stress as mentioned earlier in this review. The decrease in lymphocytes has also been seen in a similar paradigm in female rhesus macaque, where low social ranking animals showed a reduced proportion of CD8 cytotoxic cells than higher social ranking animals (Tung et al., 2012). This study holds importance as it is suggestive of similar possible consequences in human populations.

***Enrichment with novel objects and accessories.*** Though not studied as extensively as PE for immunomodulatory effects, enrichment with novel objects and accessories has been reported to primarily modify cytokine levels within the brain. Jurgens and Johnson (2012) observed reduced expression of IL-1β and TNF-α within the hippocampus, following an experimental paradigm involving exposure to novel objects and PE. This study was unable to be determined whether PE and enrichment with novel objects showed synergistic or independent effects, since the anti-inflammatory role of PE, as mentioned previously, has been well established. An independent comparative study on PE and enrichment with novel objects and accessories in the future could answer this question. In another experimental study on aged rodents, enrichment with novel objects produced a significant increase in IL-2 and TNF-α levels in the cultured supernatants of peritoneal leucocytes (Arranz et al., 2010). The authors suggested that this increase may have compensated for the age-related loss of these cytokines. However, unlike other EE studies where a number of objects, toys, and accessories were used in different combinations and changed once or twice a week, the authors in this study used only two objects at a time to maintain novelty and changed every 48 h, which could have led to handling stress and low levels of enrichment in cages. Modulation of cellular immune factors such as CD4 and CD8 T lymphocytes, and cytokines IL-2 and IL-1β by enrichment with novel objects and accessories after stressed pregnancies has also been shown in adolescent rats (Laviola et al., 2004), which suggests that novel and cognitively demanding environments can relieve stress through modulation of humoral and cellular immune factors.

***Sensory enrichment.*** Environmental enrichment studies that used sensory stimulation have not yet investigated potential immunomodulatory effects. However, research indicates that sensory stimulation can have prominent effects on the immune system. Researchers have observed enhancement in the systemic proliferation of T lymphocytes (CD4 and CD8 cells) in response to visible light passing through the eye (Roberts, 1995, 2000). A randomized trial with two experimental conditions, first watching a neutral slide show and then a disease slide show, in humans participants has shown that mere visual perception of other people’s disease symptoms can boost the immune response to microbial stimuli and increased levels of IL-6 in whole blood (Schaller et al., 2010). Visual stimuli can therefore have an important role in modulating cytokine levels in the brain, however, further research is required to establish its role in EE studies.

Immunomodulatory effects of auditory stimulation have been reported particularly in response to music in humans. Music exposure has been shown to enhance lymphocyte function in the brain, thereby reversing stress induced immunosuppression of rats during a controlled trial with two treatments, music and auditory stress (Núñez et al., 2002). Like music, group drumming therapy in age- and sex-matched human volunteers has been shown to enhance cellular immunity by increasing lymphocyte activated NK cell activity during a single trial experimental intervention with control groups (Bittman et al., 2001). Though these immunomodulatory effects in response to auditory enrichment suggest the possible association of the latter with cytokines, conclusive evidence for this association in rodent based EE studies is still not available.

Neuroimmune mechanisms associated with olfactory stimulation used as enrichment are again poorly studied, although a relationship between olfaction, autoimmunity and brain does exist and has been reviewed in detail by Strous and Shoenfeld (2006). However, the relationship between olfactory stimuli used as enrichment and immune factors in brain is not clear.

***Summary of the neuro-immunomodulatory role of other forms of EE.*** It is evident that less research has been conducted on the immunomodulatory roles of EE methods other than PE. While social enrichment could lead to immunosuppression in some rodents (Fleshner et al., 1989; Hardy et al., 1990; Stefanski and Engler, 1999; Bartolomucci et al., 2001; Stefanski, 2001), enrichment with novel objects and accessories could potentially decrease inflammation with in the brain (Jurgens and Johnson, 2012). Little work has been conducted on the immunomodulatory effects of sensory stimulation in EE studies. It is possible that a combination of different enrichment methods could provide greater enrichment to rodents and eliminate the limitations of any single enrichment method. For example, it is possible that a combination of enrichment with novel objects and accessories, and some favorable sensory stimuli could prevent immunosuppression due to social stress in a socially enriched environment.

**Table 2** presents studies that investigated the effects of EE on various cytokines and other immune factors.

**Table 2:** Effects of environmental enrichment on various cytokines and other immune parameters.

Study’s primary objective	Animal species/strain	EE methods	Frequency of changing EE method	Immune markers/behavior investigated	Significant findings	Reference
• Effect of different forms of EE on behavioral, endocrinological, and immunological parameters in male mice	• Congenic mice strain CS of the inbred strain ABG	• EH and SEH	• Once a week	• Spontaneous behavior in home cage	• ↑ Aggressive behavior in EH and SEH mice	Marashi et al. (2003, 2004)
		• EH: Standard cages with a plastic inset and wooden scaffolding		• Immunological parameters: CD4^+^ and CD8^+^ cells, cytokines (IL-2, IL-4, IL-10, and IFN-γ), IgG1 and IgG2a	• ↑ Play behavior in SEH mice. No significant differences between controls and EH mice
		• SEH: Spacious glass terraria with passable enriched cage, extra plains, plastic stairs, wooden footpaths, hemp ropes and a climbing tree. Food and water available at two places			• ↓ IgG1 in serum
					• ↑ IgG2a/IgG1
					• ↓ IFN-γ/IL-10 and IL-2/IL-10
• Effect of EE on the negative effects of influenza infection on hippocampus and spatial cognition	• 6 weeks old male Balb/c mice	• Social enrichment (five to eight per cage), toys, tunnels, ladders, housing chamber, nesting material, running wheel	• Three to four times per week	• Behavioral testing – Morris water maze	• Improved spatial learning	Jurgens and Johnson (2012)
				• Hippocampal cytokines (IL-1β, TNF-α, IL-6), chemokines (CX3CL), interferons (IFN-α and -β), and neurotrophins (BDNF and NGF)	• ↓ Expression of IL-1β and TNF-α in the hippocampus
					• ↓ Hippocampal inflammation and cognitive deficit
					• ↑ Hippocampal BDNF and CX3CL1 (anti-inflammatory chemokine) expression
• Effect of PE on protective immunity against infection	• Sprague-Dawley male rats	• Training for swimming given to rats. At the end of training period, rats were exercised by subjecting them to an exhaustive swim	–	• ELISA test for antibody response	• ↑ IgG and IgM production, T suppressor cells, and NK cells	Kaufman et al. (1994)
				• Immunofluorescent lymphocyte subtyping	• ↓ T helper cells and T helper/T suppressor ratio
• Effects of EE on several functions and oxidative stress parameters of peritoneal leucocytes in mice at different ages	• Female ICR/CD-1 mice	• Two different objects in the cages at a time	• Every 2 days at 08:00 hours	• Flow cytometry to analyze leukocyte differentiation antigens (CD11b, CD11c, CD4, CD19) and membrane expression of TLR-2 and -4	• Higher macrophage chemotactic activity and phagocytosis	Arranz et al. (2010)
		• These objects include orange bucket, jolly ball, hoop, holed ball, yellow tunnel, rough red object, yellow billiard ball, and silver ball		• Evaluation of macrophage chemotaxis, and phagocytosis	• ↑ Basal lymphocyte proliferation and chemotactic activity
		• A red kennel maintained permanently inside the cage		• Analysis of lympho-proliferation and cytotoxicity	• EE prevented age-related decline of IL-2 and TNF-α levels in old enriched mice
				• ELISA for analysis of IL-2 and TNF-α	• ↓ Expression of TLR-2 and 4 on CD4 and CD8 cells
• Effects of EE on NK cell activity, psychological stress response, and behavioral parameters	• 1 month old male C3H/eB mice	• A variety of stimuli – ladders, tunnels, and running wheels	• Once a week	• Behavioral test: Grip strength test, Elevated plus maze, and Staircase test.	• Decreased anxiety-like behavior and increased activity in Elevated plus maze and Staircase test, respectively	Benaroya-Milshtein et al. (2004)
				• Measurement of NK activity by a standard chromium release assay	• Higher NK cell activity in spleen

*EE, environmental enrichment; EH, enriched housing; SEH, super enriched housing; CD, cluster of differentiation 4; IL, interleukin; IFN, interferon; IgG, immunoglobulin G; TNF-α, tumor necrosis factor-α; CX3CL, chemokine (C-X3-C motif) ligand; BDNF, brain-derived neurotrophic factor; NGF, nerve growth factor; NK, natural killer; TLR, Toll-like receptor; ROS, reactive oxygen species; ELISA, enzyme linked immunosorbent assay.*

### EFFECTS OF EE ON GLIAL CELLS

Numbers of microglia and astrocytes, have been shown to be increased in certain regions such as the cortex and amygdala (Ehninger and Kempermann, 2003; Okuda et al., 2009) in the brains of enriched rodents during RCTs. These glial cells are known to express various cytokines and modulate the production of neurotrophins, mainly BDNF (Ferrini and De Koninck, 2013), a protein known for regulating neurogenesis in the dentate gyrus of the hippocampus (Rossi et al., 2006; Fan et al., 2007) and enhancing dendritic branching (McAllister et al., 1995; Horch and Katz, 2002; Horch, 2004). BDNF is indeed shown to enhance hippocampal neurogenesis in mice enriched with a running wheel and differently shaped objects (Rossi et al., 2006). Several other neuroglial changes have also been reported in rodents kept in an environment enriched with different methods. These include differentiation of oligodendrocyte progenitor cells into astrocytes in the amygdala of mice enriched with running wheels, tunnels and shelters (Okuda et al., 2009) and prevention of astroglial pathological changes in mice enriched with toys, nesting material, plastic houses, and tubes (Beauquis et al., 2013). Researchers also observed an increase in the expression of astrocyte GFAP (glial fibrillary acidic protein) and microglial IBA1 (ionized calcium-binding adapter molecule 1) in the dentate gyrus of rats provided with a running wheel, a polyvinyl chloride (PVC) tube and various small objects and toys (Williamson et al., 2012), and inhibition of age induced gliosis in the hippocampus of rats reared in two series of three large interconnected wire mesh cages containing various objects such as toys, balls ladders, and footbridges to play with (Soffié et al., 1999). All these changes are suggestive of the vital impact that EE has on glial cells which may in turn modulate glia-based neuroimmune mechanisms. Indeed, EE for rodents in large cages with toys and accessories and/or running wheels has shown beneficial effects in models of several brain diseases such as AD (Beauquis et al., 2013), and schizophrenia and depression (Laviola et al., 2008), which are generally associated with abnormalities in glial cells morphology and functioning (Cotter et al., 2001; Nagele et al., 2004).

**Table 3** presents studies that investigated the effects of EE on glial cells.

**Table 3:** Effects of environmental enrichment on glial cells.

Study’s primary objective	Animal species/strain	EE methods	Frequency of changing EE method	Proteins/behavior investigated	Significant findings	Reference
• Modulation of glial cells with EE in an animal model of AD	• PDAPP-J20 crossed with C57BL/6J mice	• Large cages with toys, extra nesting material, small plastic houses, and tubes. No running wheel	• Every 2 days	• Aβ peptides	• ↓ Astrocytes association with Aβ plaques	Beauquis et al. (2013)
				• Amyloid plaques	• ↓ Aβ peptides
				• GFAP^+^ glial cells	
• Effect of EE on glial cells within the hippocampus	• Adult male Sprague-Dawley rats	• A running wheel, a PVC tube, various small objects, and toys	• EE for 12 h each day in a separate cage	• Expression of various cytokines, chemokines, GFAP, and IBA1	• ↑ Astrocytes and microglia antigens expression in the hippocampus but not in CA1, CA3, and cortex	Williamson et al. (2012)
				• Expression of growth factors, BDNF and GDNF in the hippocampus.	• ↓ Expression of TNF, IL-1β, and chemokines Cc12, Cc13, and Cxc12 in the hippocampus
					• ↑ Hippocampal BDNF mRNA
• Effect of EE on the short-term memory for event durations and on the astrocytes percentage in hippocampus, frontal cortex, and corpus callosum	• Naive male Wistar derived rats 5 and 21 months of age	• Three large wire mesh cages interconnected with two tunnels, wooden and metallic objects, toys and balls, ladders, footbridges, and papers	• Daily	• Delayed symbolic matching to sample task test	• Behavioral testing resulted in ↑ in the astrocytes number and size and GFAP % in the hippocampus and corpus callosum of young rats	Soffié et al. (1999)
				• Estimation of astrocytes by evaluating GFAP percentage	• Conversely, Behavioral testing resulted in ↓ in the astrocytes number and size and GFAP % in the hippocampus and corpus callosum of old rats
					• ↓ Astrocytes number and size in the hippocampus and corpus callosum of old enriched rats
					• Reduction in memory deficit with age in old enriched rats but no total reversal of age-related impairment

*EE, environmental enrichment; AD, Alzheimer’s disease; Aβ, amyloid-β; GFAP, glial fibrillary acidic protein; PVC, polyvinyl chloride; IBA1, ionized calcium-binding adapter molecule 1; BDNF, brain-derived neurotrophic factor; GDNF, glial cell-derived neurotrophic factor; CA, Cornu Ammonis; Cc12, (C–C motif) ligand 12; Cc13, (C–C motif) ligand 13; Cxc12, (C–X–C motif) ligand 12; mRNA, messenger ribonucleic acid.*

## DISCUSSION

### IMMUNOMODULATION BY DIFFERENT METHODS OF ENVIRONMENTAL ENRICHMENT

**FIGURE 4:**
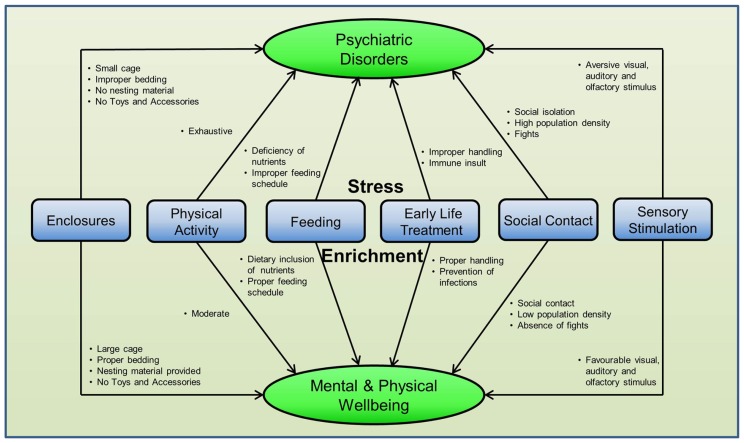
**Differential effects of enrichment methods in rodents.** The same stressful factor when subtly modified can become enrichment for the animals in captivity. This suggests that environment for rodents can be enriched by changing the existing arrangement of different factors around them and no special efforts are required.

Provision of voluntary wheel running, social housing, cognitive training, and sensory stimulation may act as mild stressors in the initial stages but a number of studies have shown constructive neurobiological and behavioral variations in response to these enrichment methods, particularly to PE and enrichment with novel objects and accessories. Ironically, the same causal factors for stress when altered finely can become favorable for living and can be used for enrichment during psychiatric disorders. See **Figure 4** for more details.

The neuroimmunomodulatory role of PE has been extensively studied and appears to be the strongest form of enrichment when used alone in both rodents and human studies. PE can modulate a number of brain regions which may in turn result in varied functional outcomes, such as improvement in memory (Erickson et al., 2011), learning (Van Praag et al., 2005), anxiety- and depressive-like behaviors (Binder et al., 2004; Zheng et al., 2006; Duman et al., 2008; Marais et al., 2009), cognition (Griffin et al., 2009; Nichol et al., 2009), and motor activity (Biernaskie and Corbett, 2001), and this has been highly regarded by researchers in their publications. PE mostly affects the humoral immune system; however, its role in the modulation of cellular immune system cannot be ignored. Post-exercise, production and expression of anti-inflammatory factors, particularly anti-inflammatory cytokines (e.g., IL-6, IL-10) are enhanced in both the systemic circulation as well as within the brain. This subsequently reduces the level of pro-inflammatory factors, such as the cytokines TNF-α and IL-1β (Eyre and Baune, 2012), chemokines (Ostrowski et al., 2001), TLRs (Gleeson et al., 2006), and CRP (Koletzko, 2003), helping in alleviating both systemic and neuroinflammation, the latter being the causal factor for most psychiatric disorders. An increase in the number of T lymphocytes and NK cells after PE (Kaufman et al., 1994) strengthens adaptive immunity. Modulation of glial cells, T cells, and macrophages in the brain by PE also helps in reducing the neurotoxic effects and enhances neurogenesis in the brain, particularly in the hippocampus (Eyre and Baune, 2012). Though moderate PE has been reported to induce beneficial effects, exhaustive PE has been shown to result in immunosuppression in human participants (Mars et al., 1998; Tuan et al., 2008) which suggests that voluntary wheel running is probably more useful for inducing favorable neuroimmune changes than forced exercise on a treadmill. The latter could cause stress to rodents in EE studies. Further, external environmental conditions (e.g., heat, cold, humidity) could play a role in the immunomodulatory effects of PE on the brain, as stated earlier in this review. While rodents are reared in standard environmental conditions with all variables controlled throughout the life span of rodents, the same may not be applicable to humans.

Social and cage enrichment are the simplest avenues to modulate behavior, however, formation of dominant and subordinate populations can affect the response, with detrimental effects seen mainly in subordinate animals. The latter have shown signs of immunosuppression and depression in enrichment studies which clash with the principles of enrichment, i.e., making the environment favorable for living. In fact, cellular analyses have revealed loss in the number and function of splenocytes, decreased anti-inflammatory cytokines (e.g., IL-10), T cells, NK cells, and serum antibodies in subordinate animals (Fleshner et al., 1989; Bartolomucci et al., 2001). Several EE studies have used social enrichment for rodents (Angelucci et al., 2009; Jurgens and Johnson, 2012); however, they have not reported on the presence or absence of dominant and subordinate, which might have confounded the findings from these studies.

“Novelty seeking” behavior is the inherent tendency to explore novel objects and accessories, and has been investigated in many enrichment studies. Novel objects and accessories used in conjunction with PE have been reported to reduce the expression of IL-1β and TNF-α in the hippocampus (Jurgens and Johnson, 2012) suggestive of anti-inflammatory effects. This, however, makes it difficult to conclude whether these anti-inflammatory effects were seen in response to PE and/or to the novel objects and accessories. Nevertheless, the role of novel objects in cell-mediated immunity cannot be disregarded as improvement in macrophage chemotaxis and phagocytosis, lymphocyte chemotaxis, and NK with two novel objects at a time (Arranz et al., 2010). It is possible that changes in the methods of enrichment and rearrangement of objects in space and time are required for the sustained beneficial effects on the brain of the complex environment devoid of running wheels; but substantial evidence is still required to establish this hypothesis. Some EE studies failed to mention whether the objects were changed (Nakamura et al., 1999; Kempermann et al., 2002; Lazarov et al., 2005). If the objects were not replaced regularly to maintain novelty, it may have affected the immune response and behavior of rodents during study. Furthermore, few studies have investigated the immunomodulatory effects of enrichment with novel objects, and no meta-analysis is available to verify the results at this stage, thus making it essential to validate these findings with more extensive research. In terms of the human environments, a subject receives several kinds of stimulus in addition to PE, which could have confounding effects on modulation of brain function.

The immunomodulatory mechanisms associated with sensory enrichment have not been investigated in EE studies. Sensory enrichments have been shown to enhance sensory functions (visual, auditory, or olfactory), as well as improve cognition and behavior (see review by Wells, 2009), although the neuroimmune mechanisms accountable for this improvement in brain functions are not fully described and therefore this needs further attention in future studies.

It appears that EE is a very complex process and that a standard rodent environment may also involve all methods of enrichment at the same time to some extent. For instance, rodents climbing the cage walls/grid could be an example of resistance exercise which is seen even in the absence of a running wheel in cages and has been reported to improve spatial memory (Cassilhas et al., 2012). Similarly, resistance exercise in healthy humans (knee extension under alternating concentric and eccentric conditions for muscle work), in the absence of any endurance exercise, improved serum concentrations of insulin-like growth factor 1 (IGF-1), which has been shown to mediate positive effects of exercise on brain functions (Carro et al., 2000; Vega et al., 2010). Further, social enrichment through housing in pairs could lead to the formation of a dominant and submissive mouse (Malatynska and Knapp, 2005). In addition, it is possible that the sight of a novel object, auditory stimulus during handling or changing cages, and olfactory cues from handler or an injured mouse in a social environment could modify immune parameters in the brain, and in turn neurobiology and behavior. Likewise, any new nesting material or sensory stimuli could also possibly stimulate “novelty seeking” behavior of rodents. Therefore, this necessitates thorough examination of all external environmental variables in EE paradigms.

No research to date has used standardized enrichment techniques and different methods have been used randomly. This standardization of enrichment techniques is necessary since different methods of EE can elicit diverse effects on neurobiology, behavior, and neuroimmune mechanisms. Moreover, a standard human environment is similar to an enriched environment for rodents. The results from enrichment through PE in rodents can successfully be translated to human intervention for psychiatric illnesses; however, findings from other enrichment methods in rodents may be of less value in translating the effects to humans. An intense examination of human nature and its application while formulating an environment enriched with cognitively stimulating activities is essential for similar interventions in humans.

### A NOTE OF CAUTION ON THE USE OF NUTRITIONAL ENRICHMENT

While some researchers have advocated the use of food for enrichment to improve behavior (Harris et al., 2001; Brown, 2009), others have avoided using nutritional enrichment apparently due to the confounding effects of various nutrients on neurobiology and behavior when used in conjunction with other enrichment methods. Evidently, diets rich in essential fatty acids (EFA), such as omega-3, normalize the levels of brain proteins, reduce oxidative stress, maintain neuronal plasticity, improve mood, and enhance learning and cognitive abilities (Richardson, 2003; Wu et al., 2004) in both rodents and humans. Such subjects are also less susceptible to stress and show improved behavior and enhanced memory (Wainwright et al., 1994; Wainwright, 2002; Fedorova and Salem, 2006; Uauy and Dangour, 2006). Similarly, a high protein and glucose diet has been shown to enhance the growth of the brain and its functions (Diamond, 2001). The effects of anti-oxidants, anti-inflammatory components, vitamins, and minerals in food on behavior, learning, and cognition have been studied and reviewed extensively in the past (Wasantwisut, 1996; Beard, 2003; Navarro et al., 2005; Buell and Dawson-Hughes, 2008; Joseph et al., 2009; Ford et al., 2010; Kennedy and Haskell, 2011). Yet, the effects of food variety are difficult to distinguish from the intrinsic nutritional effects of the specific food that is used for enrichment. It is likely that food used in combination with other enrichment methods could have confounded the neurobiological and behavioral effects depending on its nutrient composition in EE studies.

### TIME OF ENRICHMENT IN THE LIFE OF AN ANIMAL

Besides different methods of EE, the stage of life when enrichment has been given can potentially affect results. During neonatal and early prenatal periods, the brain develops rapidly, large numbers of new synapses are formed and growth and differentiation of the cerebro-cortical region takes place. Any changes in the brain induced during this period can persist throughout life. Environmental conditions during prenatal and early stages of the life cycle can have distinct effects on neurobiology and behavior (Chapillon et al., 2002; Gutman and Nemeroff, 2002). Moreover, studies have shown that immune insult during pregnancy can affect growth and behavior of offspring and makes them susceptible to mental disorders such as AD, schizophrenia, and autism (Shi et al., 2003; Bakos et al., 2004; Brown, 2006).

While prenatal stress has been shown to suppress NK cell cytotoxicity and reduce B cell proliferation in an experiment on rats (Kay et al., 1998), a review suggests that perinatal infection can cause long-term alteration in cytokine production and brain glial cell function and is generally manifested by marked cognitive and behavioral changes throughout the lifespan (Bilbo and Schwarz, 2009). When exposed to perinatal infection and neonatal maternal separation in controlled trials, rodents displayed marked cognitive and behavioral changes, and impaired learning and memory which persisted throughout their lifespan (Bilbo and Schwarz, 2009; Vivinetto et al., 2013). Loss of hippocampal plasticity (Mirescu et al., 2004) and sex-specific changes in hippocampal dendritic complexity and dendritic spine density (Bock et al., 2011) has also been reported in adult rats exposed to early life stressful experiences. This suggests that providing enrichment during early stages of the life cycle can help to safeguard against psychiatric disorders in later life. Indeed, neonatal handling with an enriched environment can reduce the signs of emotionality and anxiety, augment novelty seeking behavior, and can have preventative actions on age-related learning impairments and hippocampal neuronal atrophy in rodents (see review by Fernandez-Teruel et al., 1997). Likewise, enrichment with frequently changed toys after weaning provided a beneficial intervention for reversing the harmful effects of maternal separation in rats (Francis et al., 2002). This decreased reactivity to stressful stimuli, however, was later found to be the function of a less sensitive HPA axis (Anisman et al., 1998; Welberg et al., 2006). Contrary to these findings, Papaioannou et al. (2002) observed sexual differences and suggested that neonatal handling increases the capacity of male Wistar rats to face chronic stressors, and increases the susceptibility to express “depressive” behavior in female. The authors have attributed this discrepancy between the two sexes to the combination of decreased serotonergic activity with high circulating corticosterone levels in female rats.

Controlled trials on Sprague-Dawley rats have established that enrichment in early life increases T cell numbers, enhances production of anti-inflammatory cytokines (e.g., IL-2, IL-10) and lowers production of the pro-inflammatory cytokine IL-1β in various brain regions such as the hypothalamus and frontal cortex (Laviola et al., 2004; Bilbo et al., 2007), suggesting attenuating effects of early life enrichment on neuroinflammation. This suggests that requisite neurological and behavioral enhancements can be more readily achieved by enriching the environment of an animal at the prenatal stage and preserving it later in life with regular novel and enriching inputs.

### SIGNIFICANCE OF IMMUNE AND NON-IMMUNE FACTORS IN ENVIRONMENTAL ENRICHMENT PARADIGMS

It is evident that overexpression of pro-inflammatory cytokines and chemokines in the brain, in addition to decreases in cytotoxic T cell proliferation and activity may result in diminished cognitive performance and development of neuropathology (Arvin et al., 1996; Hawkley and Cacioppo, 2004; Baune et al., 2008). These cytokines are primarily expressed by glial cells in the brain whose levels increase with aging. However, the neuronal aging slows down in mice raised in enriched environment, and is characterized by sustained neurogenesis and reduced neuronal damage in the cellular microenvironment of the dentate gyrus (Nilsson et al., 1999; Gould et al., 2000; Kempermann et al., 2002). Indeed, EE has been shown to improve the plasticity of cognitive functions and learning performance, and reduce the impairment of spatial memory in aged rodents (Kobayashi et al., 2002; Frick and Fernandez, 2003; Frick et al., 2003). In addition, the role of cellular immunity is important when studying immune effects of external environmental stimuli (Stefanski and Engler, 1999; Stefanski, 2001; Benaroya-Milshtein et al., 2004).

Several non-immune factors such as neurotransmitters and neurotrophins have also been implicated in the neural changes within the enriched environment. It has been suggested that neurotransmitters, such as dopamine, serotonin, and GABA, mediate communication between the nervous system and immune system (Mössner and Lesch, 1998; Basu and Dasgupta, 2000; Felten, 2008; Bhat et al., 2010) and their levels change in different brain regions of rodents, such as the hippocampus and prefrontal cortex, in response to an enriched environment (Mora et al., 2007). Indeed, in a study on aged rats kept in an environment enriched with running wheels, a re-arrangeable set of plastic tunnels, an elevated platform and toys, the extracellular levels of the neurotransmitters GABA and glutamate showed an increase in the CA3 area of the hippocampus (Segovia et al., 2006). Likewise, levels of neurotrophins, nerve growth factor, BDNF, and neurotrophin-3, have been shown to be increased in different brain region of rats, such as the cerebral cortex, hippocampus, and forebrain after treatment with EE comprising of running wheels, toys, and novel objects (Ickes et al., 2000; Pham et al., 2002; Gobbo and O’Mara, 2004; Angelucci et al., 2009). Indeed, the increase in hippocampal expression of BDNF in response to voluntary wheel-running (Duman et al., 2008) and 1-week forced treadmill exercise (Griffin et al., 2009) in rodents has been attributed to improvement in cognitive functions and reduction in anxiety- and depressive-like behaviors by some authors.

Taken together, the finding in this review, suggest that improvement and development of the environment is beneficial to preserve and enhance species-typical behavioral aspects by altering immune parameters in association with genes and neuro-chemicals. However, the immunomodulatory roles of enrichment methods other than PE have received less attention and a better understanding is required.

## LIMITATIONS OF THIS REVIEW

The primary aim of this review is to discuss the neuro-immunomodulatory mechanisms that govern effects of various EE methods on brain functions such as cognition and memory. Since most EE studies have been conducted on rodents, the studies included in this review investigated the role of EE in modulating neurobiology and behavior, and associated immune mechanisms in rats and mice. As such, all evidence reported may not provide similar results in other animals or in humans. However, this review provides the foundation to model similar or equivalent enrichment techniques in other species of animal, as well as portray the possible consequences of enriching the existing environment for humans, though the outcome may vary depending on the existing circumstances of each individual. This is contrary to rodents in cages when all animals are in similar environmental conditions during study. Moreover, the limited number of studies on the immunomodulatory effects of EE methods in rodents, other than exercise, also limited our efforts to include all immune factors, for example, CRP, MAPK, and the complement system, under consideration for each enrichment method and compare them with similar results in human intervention. This suggests that though this review provides a comprehensive account for EE effects of neuroimmune modulation, extensive research is still needed to establish that EE provides beneficial intervention for psychiatric disorders and neuropathological conditions via modulation of cytokines and other humoral and cellular immune factors.

## CONCLUDING REMARKS

There are many complexities involved in EE paradigms, as clear from our discussion in this review. Although it is widely accepted that enriching the environment exerts distinct beneficial effects on the learning and memory competence of an animal and therefore, considering various environmental factors is vital while formulating EE methodology for studies on its effects on brain functions. Substantial evidence confirms that PE alleviates psychiatric disorders via modulation of neuroimmune mechanisms (Kaufman et al., 1994; Pedersen and Hoffman-Goetz, 2000; Eyre and Baune, 2012), however, the same cannot be said conclusively for other enrichment methods. While the immunomodulatory mechanisms of EE in controlling brain diseases and cognitive disorders in rodents have received much attention in the last decade, similar studies in humans to investigate the immunomodulatory effects of analogous EE methods (as pointed out earlier in this review) in psychiatric disorders need to be conducted. Since physical activity is a form of EE in rodents and reduces cognitive and memory deficits, the hypothesis that a combination of PE and cognitive training will have a preventative and therapeutic effect on human brain disorders via anti-neuroinflammatory and anti-neurodegenerative mechanisms, still needs to be investigated. Additionally, a study on combined effect of PE with other EE methods and/or pharmacological drugs on a long-term and short-term basis in rodents will be helpful to develop new, and optimize current, immunomodulatory preventative and treatment therapies for cognitive dysfunction and associated brain disorders.

## Conflict of Interest Statement

The authors declare that the research was conducted in the absence of any commercial or financial relationships that could be construed as a potential conflict of interest.
